# Acute motor deficit and subsequent remyelination‐associated recovery following internal capsule demyelination in mice

**DOI:** 10.1111/jnc.15142

**Published:** 2020-08-14

**Authors:** Reiji Yamazaki, Nobuhiko Ohno, Jeffrey K. Huang

**Affiliations:** ^1^ Department of Biology and Center for Cell Reprogramming Georgetown University Washington DC USA; ^2^ Division of Histology and Cell Biology Department of Anatomy School of Medicine Jichi Medical University Shimotsuke Japan

**Keywords:** demyelination, internal capsule, lysophosphatidylcholine, motor recovery, remyelination

## Abstract

Multiple sclerosis is a chronic inflammatory demyelinating disease of the central nervous system (CNS), characterized by accumulated motor disability. However, whether remyelination promotes motor recovery following demyelinating injury remains unclear. Damage to the internal capsule (IC) is known to result in motor impairment in multiple sclerosis and stroke. Here, we induced focal IC demyelination in mice by lysophosphatidylcholine (LPC) injection, and examined its effect on motor behavior. We also compared the effect of LPC‐induced IC damage to that produced by endothelin‐1 (ET1), a potent vasoconstrictor used in experimental stroke lesions. We found that LPC or ET1 injections induced asymmetric motor deficit at 7 days post‐lesion (dpl), and that both lesion types displayed increased microglia/macrophage density, myelin loss, and axonal dystrophy. The motor deficit and lesion pathology remained in ET1‐injected mice at 28 dpl. In contrast, LPC‐injected mice regained motor function by 28 dpl, with corresponding reduction in activated microglia/macrophage density, and recovery of myelin staining and axonal integrity in lesions. These results suggest that LPC‐induced IC demyelination results in acute motor deficit and subsequent recovery through remyelination, and may be used to complement future drug screens to identify drugs for promoting remyelination.

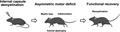

## Graphical abstract

In this issue, we examined the effect of focal internal capsule (IC) demyelination on motor behavior in mice. We found that lysophosphatidylcholine (LPC) induced demyelination at the IC resulted in acute asymmetric motor deficit, followed by subsequent remyelination‐associated functional recovery. Our results suggest that IC demyelination is a tractable model for characterizing demyelination and remyelination through behavioral measurements, and may be used to complement future drug discovery efforts for promoting repair in inflammatory demyelinating disorders, such as multiple sclerosis (MS). Images generated with BioRender.

**Abbreviations**
APC/CC1: adenomatous polyposis coli
dpl: days post lesion
ET1: endothelin‐1
FM: FluoroMyelin
Iba1: inonized calcium‐binding adapter molecule
IC: internal capsule
iNOS: inducible nitric oxide synthase
LFO: left forepaw only
LH: left hindlimb
LPC: lysophosphatidylcholine
MBP: myelin basic protein
MS: multiple sclerosis
NF: neurofilament
NR: neutral red
OLs: oligodendrocytes
OPCs: oligodendrocyte precursor cells
PDGFRα: platelet‐derived growth factor receptor α chain
RFO: right forepaw only
RH: right hindlimb
WM: white matter

## 1 INTRODUCTION

Multiple sclerosis (MS) is a chronic inflammatory demyelinating disease of the CNS characterized by progressive remyelination failure, axonal loss, and the accumulation of clinical disability (Compston & Coles, 2008; Dutta & Trapp, 2014; Lassmann, Horssen, & van, Mahad D., 2012; Reich, Lucchinetti, & Calabresi, 2018). Rodent models of demyelination are frequently used to study the mechanisms of remyelination and to investigate potential treatments for MS. These models include focal demyelination by the injection of lysophosphatidylcholine (LPC) into the spinal cord or corpus callosum (Blakemore & Franklin, 2008; Keough, Jensen, & Yong, 2015), or cuprizone diet intoxication, which induces corpus callosum demyelination (Matsushima & Morell, 2001; Sachs, Bercury, Popescu, Narayanan, & Macklin, 2014). While these models are highly informative for the evaluation of oligodendrocyte lineage cell progression and remyelination after demyelination, clinical symptoms such as motor dysfunction and paralysis are not usually observed. Another demyelination model, experimental autoimmune encephalomyelitis (EAE), is mainly used to evaluate immune‐mediated demyelination pathology and the development of progressive motor deficit (Constantinescu, Farooqi, O’Brien, & Gran, 2011; Robinson, Harp, Noronha, & Miller, 2014). However, remyelination analysis in the EAE model is challenging, as lesions of varying status are widely distributed throughout the spinal cord (Baydyuk et al., 2019) and recovery from motor deficit does not usually occur. As such, a tractable rodent model to determine if remyelination promotes functional recovery remains unavailable.

Damage to the internal capsule (IC), the major trajectory of motor neurons from the cerebral cortex to the spinal cord, is known to induce motor deficit. It is frequently observed in white matter stroke (Schiemanck, Kwakkel, Post, Kappelle, & Prevo, 2008; Shelton & Reding, 2001), and has also been observed in MS (Lee et al., 2000; Maimone, Reder, Finocchiaro, & Recupero, 1991). Experimental cerebral infarction at the IC can be induced by administering endothelin‐1 (ET1), a strong vasoconstrictor that induces fibrosis, hypertrophy, inflammation, oligodendrocyte, and axonal loss (Kedzierski & Yanagisawa, 2001; Ono, Imai, Miyawaki, Nakatomi, & Saito, 2016; Roome et al., 2014; Tennant & Jones, 2009) that result in lasting motor deficit (Ahmad, Satriotomo, Fazal, Nadeau, & Doré, 2015; Blasi, Whalen, & Ayata, 2015). However, whether demyelination at the IC induces motor deficit and potential recovery through myelin repair has not been demonstrated.

Here, we examined motor behavior in mice following LPC injection at the IC, and compared its effect to mice that received ET1‐induced IC infarct. We found that unilateral LPC‐induced IC lesion resulted in the development of asymmetric motor deficit similarly to that observed with ET1 lesion at 7 days post lesion (dpl). However, in contrast to ET1‐lesioned mice, which do not recover from motor deficit, LPC‐lesioned mice eventually regained motor function by 28 dpl. Furthermore, LPC‐lesioned mice displayed increased oligodendrocyte density, reduced axonal dystrophy, and recovery of myelin staining at the IC lesion at this timepoint. Together, our results suggest that LPC‐induced IC demyelination induces acute behavioral deficit, which is followed by subsequent functional recovery through remyelination. Moreover, focal IC demyelination may a tractable model for the evaluation of functional recovery through remyelination, providing a complement to future drug discovery efforts for promote repair in MS.

## 2 MATERIALS AND METHODS

### 2.1 Animals

**Figure 1:**
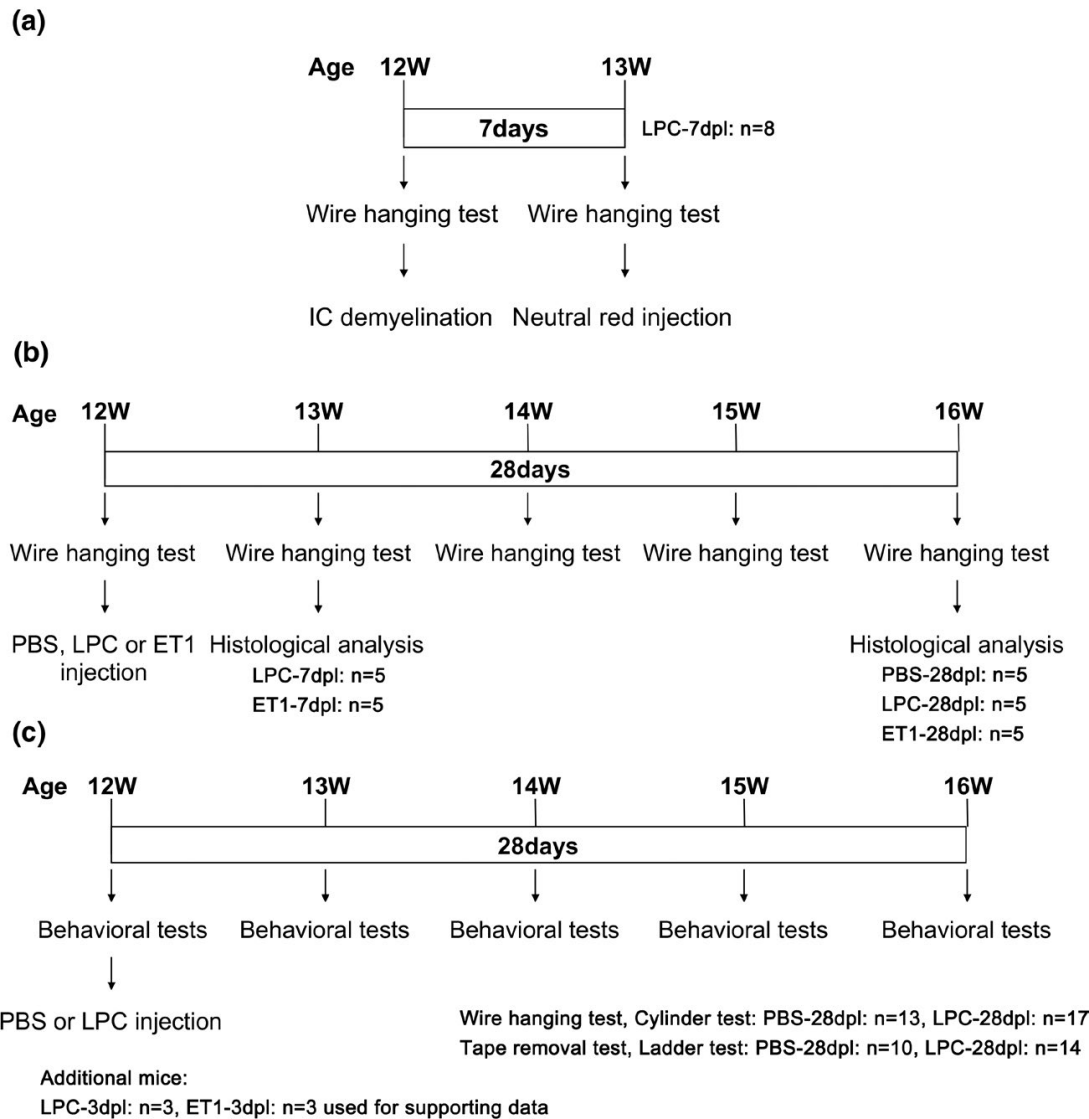
Schematics of experimental design and timeline in this study. (a) Schematic diagram of wire‐hanging test of internal capsule (IC)‐demyelinated mice for Figure 2 data. (b) Schematic diagram of motor function and histological analysis between lysophosphatidylcholine (LPC) and endothelin‐1 (ET1)‐induced IC injuries for Figures 3–6. (c) Schematic diagram of behavioral changes by IC demyelination for Figure 7

This study was not pre‐registered. To perform IC lesion experiments, 12‐week‐old male C57BL/6J mice (20–30 g) (RRID:IMSR_JAX:000664) were purchased from Charles River (82 mice) or Japan SLC (6 mice) and maintained in the animal facility of the Georgetown University or Jichi Medical University. Experiments were performed in the United States and Japan. These mice were kept in standard cages with fewer than five mice per cage at 20–25°C on a 12‐hr light/dark cycle. All animal experiments were performed in accordance with the ARRIVE (Animal Research: Reporting In Vivo Experiments) guidelines. All efforts were made to use only the number of animals necessary. All experiments were approved by the Institutional Animal Care and Use Committee (IACUC) at the Georgetown University (approval number 2016‐1123) and Jichi Medical University (approval number 19034‐02) and performed in accordance with the guidelines on the care and use of animals of this committee. Experiments were exploratory in nature, and initially eight mice were used to test the effect of LPC IC lesions on motor behavior and neutral red (NR) detection. For statistical analyses, 10–15 mice were needed to evaluate behavioral changes, based on previously published work (Martinez‐Huenchullan et al., 2017; Rabl et al., 2016; Roome & Vanderluit, 2015). The number of mice used was also based on their availability and age in our animal vivarium at the time of the surgeries. The animal groups and experimental timelines are shown in Figure 1. A total of 88 mice (PBS = 23 mice, LPC = 47 mice, ET1 = 18 mice) were used in this study for histology and behavioral tests (Table S1). Two mice that received ET1 were excluded because they died before completion of the behavioral analysis, and were not replaced. To reduce mouse suffering, no more than two behavioral tests were performed in one day. All experiments were conducted between 9 a.m. and 7 p.m.

### 2.2 Immunohistochemistry

Mice were anesthetized through 3% isoflurane inhalation for rapid induction and to minimize suffering before transcardial perfusion with 2 ml/g body weight of 4% (w/v) paraformaldehyde (PFA) in phosphate‐buffered saline (PBS), pH 7.4. After perfusion, brain tissues were immersed overnight in 4% PFA/PBS at 4°C for post‐fixation of tissue. For cryoprotection, fixed brains were immersed overnight in 15% (w/v) sucrose (Cat# S0389, Sigma‐Aldrich) at 4°C, followed by another overnight immersion in 30% (w/v) sucrose (Cat# S0389, Sigma‐Aldrich) at 4°C, before freezing in OCT compound (Sakura Finetec) on the dry ice. Cryosections (12 µm thick) were prepared using a cryostat (CM1900; Leica Microsystems). To identify brain sections containing the internal capsules, toluidine‐blue staining was used before sections were collected on SuperFrost Plus slide (VWR International), and stored at −80°C. For FluoroMyelin staining of cryosections were incubated with FluoroMyelin Red (RRID:AB_2572213, Cat# F34652, Thermo Fisher scientific) in 1XPBS, pH 7.4, containing 0.1% Triton X‐100 (TX) for 40 min at room temperature or 20°C. For immunofluorescence staining, cryosections were permeabilized and blocked in blocking buffer (0.3% TX‐TBS and 10% goat serum) for 1 hr, incubated overnight at 4°C with primary antibodies in blocking buffer, washed three times in 0.1% TX‐TBS, incubated for 1 hr at room temperature or 20°C with secondary antibodies, and washed three times in 0.1% TX‐TBS before mounting (Yamazaki, Baba, & Yamaguchi, 2018). These images were captured by a confocal microscopy (LSM 880; Zeiss or FV1000; Olympus).

### 2.3 Focal demyelination of the posterior internal capsule

Demyelination was induced by injection of 1% lysophosphatidylcholine (LPC) (Cat# L4129, Sigma‐Aldrich) in PBS into the internal capsule. Mice were anesthetized by ketamine/xylazine cocktail (100 mg/kg and 10 mg/kg, respectively) and placed on the stereotaxic frame (Narishige) using mouse ear bar. Ketamine/xylazine was used to maintain sedation throughout the duration of the surgery without the need for continuous anesthetic flow (i.e., with isofluorane) via a nose piece on the stereotaxic frame. To reduce pain associated with the surgery and to minimize animal suffering, mice were treated with the analgesic, carprofen (5 mg/kg) once before and once 24 hr after surgery. Bupivacaine (0.05–0.1 ml of 25%) was delivered once intra‐incisionally at the site of injection to reduce pain locally. PBS, LPC or ET1 (Cat# E7764, Sigma‐Aldrich; 0.1 mg/ml) were stereotaxically injected into the right internal capsule (anteroposterior −1.5 mm from the bregma, mediolateral +2.5 mm from the midline, dorsoventral −4.0, −3.5 and −3.0 mm from the dura). Hamilton syringe (Cat# 24530; Sigma‐Aldrich, 31 gauge) connected to the micro injector (IMS‐3; Narishige) was inserted into the internal capsule and was kept for 3 min before injection of PBS or LPC or ET1 (total 3 µl volume; injection of 1 µl volume at the depth of 4.0, 3.5 and 3.0 mm, respectively). Each position injection took interval for 2 min. The needle was kept for 10 min after injection to reduce reflux along the needle track. After injection and needle removal, the overlying skin was sealed with a surgical adhesive (Vetbond). The mice were then sacrificed at 3, 7, and 28 dpl.

### 2.4 Antibodies

The primary antibodies for immunofluorescence staining were rabbit polyclonal anti‐ionized calcium‐binding adapter molecule 1 (Iba1) (RRID:AB_839504, Cat# 019‐197411:400; Wako), rabbit polyclonal anti‐neurofilament (NF) 200 (RRID:AB_477272, Cat# N4142, 1:200; Sigma‐Aldrich), rabbit polyclonal anti‐Olig2 antibody (RRID:AB_1630817, Cat# 18953, 1:100; IBL America), rabbit polyclonal anti‐Type I collagen (RRID:AB_1962715, Cat# LB‐1102, 1:1,000; LSL), rat monoclonal anti‐myelin basic protein (MBP, aa‐82‐87) (RRID:AB_94975, Cat# MAB386, 1:200; Millipore), rat monoclonal anti‐platelet‐derived growth factor receptor α chain (PDGFRα) (RRID:AB_397117, Cat# 558774, 1:100; Bioscience), mouse monoclonal anti‐adenomatous polyposis coli (APC/CC1) (RRID:AB_2057371, Cat# OP80, 1:100; Merck Millipore), mouse monoclonal anti‐NF H non‐phosphorylated mouse mAb (SMI 32) (RRID:AB_2043449, Cat# NE1023, 1:200; Calbiochem), mouse monoclonal anti‐inducible nitric oxide synthase (RRID:AB_397719, Cat# 610329, 1:100; BD Biosciences) antibodies. Alexa Fluor anti‐rabbit 488‐ (RRID:AB_143165, Cat# A‐11008), anti‐rat 594 (RRID:AB_10561522, Cat# A‐11007) or anti‐mouse Texas Red‐ (RRID:AB_2556778, Cat# T‐6390) conjugated species‐specific antibodies (1:1,500; Thermo Fisher Scientific) with 1 µg/ml Hoechst 33342 (Cat# 3570; Thermo Fisher Scientific) for labeling nuclei.

### 2.5 Neutral red labeling of demyelinated lesion

Following IC damage, 500 µl of 1% neutral red (Cat# N4638, 10 mg/ml; Sigma‐Aldrich) in PBS was injected by intraperitoneally (IP) injection for each mouse as described previously (Baydyuk et al., 2019). After 2 hr, mice were transcardially perfused with 1 ml/g body weight of PBS. For fixation of LPC‐injected brain, dissected tissues were immersed overnight in 4% PFA/PBS at 4°C. After fixation, coronal section of the brain containing internal capsules was generated to identify neutral red labeling. Neutral red‐labeling images were captured by a light microscopy (MVX10; Olympus, AxioCam ERc5s; Zeiss).

### 2.6 Behavioral tests

An overview of the behavioral test timeline is shown in Figure 1. The cylinder test was performed to assess preferred forepaw use during exploratory behavior (Blasi et al., 2015; Starkey et al., 2005). Mice were placed in the cylinder (20 cm in height, 10 cm in diameter) for 7 min. A video camera was placed at the bottom of the cylinder to record behavior. The number of wall touches for weight support with right, left, and both forepaws was counted. The percentage of each forepaw usage were calculated in each session.

The adhesive tape removal test was performed to assess forepaw sensorimotor function as described previously (Blasi et al., 2015; Bouet et al., 2009; Tennant & Jones, 2009). Briefly, a small piece of adhesive tape (Cat# 15‐901, 10 × 5 mm; Fisher) was placed on the surface of the right or left forepaw. The sensorimotor score is the total time it took from the mouse noticing the tape on its forepaw (by putting its mouth to the tape or shaking its paw) to when the tape is removed from its forepaw with its mouth or through shaking. Three training sessions per day for 3 days were performed before surgery. For scoring, three trials per session were performed, and the scores were recorded and averaged.

The horizontal ladder test was performed to assess forelimb and hindlimb motor function (Farr, Liu, Colwell, Whishaw, & Metz, 2006; Metz & Whishaw, 2002). A ladder of 120 cm and 5‐cm wide (Cat# 76‐0931; Harvard Apparatus) was placed from a start cage and to the home cage. Mice were placed on the start cage of the ladder and walked toward the home cage. Three training sessions per day for 3 days were performed before surgery. After training, three walking sessions were performed for each trial. The waking performance was recorded by a video camera from the side of the ladder. The total foot slips numbers for each walk were recorded, and the average slip numbers per trial were calculated.

The wire‐hanging test was performed to evaluate motor balance, co‐ordination, and muscle condition (Aartsma‐Rus & van Putten, 2014; Dorchies et al., 2013). A 2‐mm‐thick metal hanger was placed to a shelf with tape. The mouse being examined is first placed on the wire through both of its forepaws. The mouse will proceed to lift itself up through its tail or hindlimb and stay on the wire before losing grip/balance and falling off the wire onto soft bedding. Hanging time corresponds to the start of wire placement to the time the mouse falls off the wire onto soft bedding. The limit of hanging time was 5 min after the timer starts. If the mouse falls off before 5 min, it will be placed back on the wire up for up to two more times. The longest hanging time during the 5‐min trial was recorded over three sessions and averaged for each group. Mice with significant motor deficit was defined as displaying decreased longest hanging time by more than 60 s compared to before injury, whereas those with mild motor deficit was defined as displaying decreased longest hanging by 30 s to 60 s. No motor deficit was defined as mice with increased or decreased longest hanging time by less than 30 s.

For behavioral task scoring, mice were arbitrarily chosen, without specific randomization methods. Each mouse was assigned based on cage number and permanent marker numbering on the tail without knowledge of the experimental injury condition, and scores were recorded at specified timepoints until 28 dpl. The experimenter was unaware of the animal's injury group during behavioral analysis, and during statistical analysis. Mouse identities and injury condition were revealed only after all raw values (i.e., time, paw usage, or foot slips) were recorded and analyzed statistically.

### 2.7 Statistical analysis

All quantitative analyses are presented as mean ± standard deviation (*SD*) from each mouse. For quantification, the areas and fluorescent intensities of images captured by confocal microscopy were determined by Fiji‐imageJ. Statistical analyses were performed using Prism 7 (GraphPad Software). No sample size calculation was performed. No exclusion criteria were pre‐determined, and no data points were excluded. Statistical comparisons were performed using a Student's *t* test, one‐way ANOVA or two‐way ANOVA followed by a post hoc Tukey–Kramer test, which assumes a normal distribution. We performed one‐way ANOVA to analyze all immunostaining data (Figures 4, 5, 6), and a two‐way ANOVA in all behavioral analysis (Figures 3 and 7). We quantified microglia/macrophage accumulated‐area (mm^2^) from Iba1 immunostaining in the lesioned internal capsule (Figure 3d). The significance was indicated as a *p* value: *p < .05, **p < .01 and ***p < .001 for immunostaining analysis; ^#^ *p* < .05, ^###^ *p* < .001 for behavioral analysis.

## 3 RESULTS

### 3.1 Analysis of internal capsule (IC) demyelination versus. stroke injury in mice

**Figure 2:**
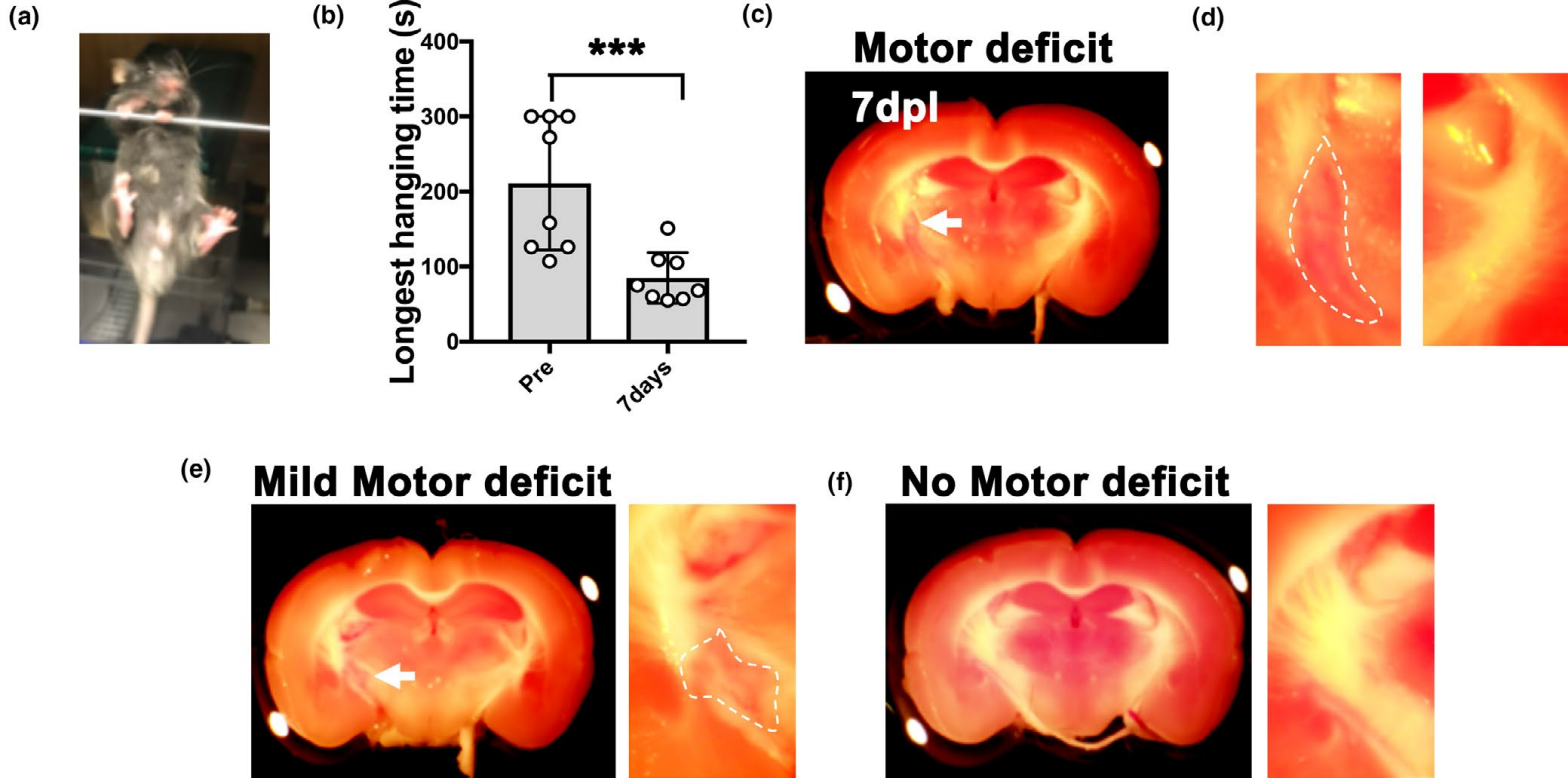
Behavioral analysis of internal capsule (IC)‐demyelinated mice by wire‐hanging test. (a) Start position of the wire‐hanging test. (b) Quantification of the longest hanging time before lesion (Pre) and at 7 days after lysophosphatidylcholine (LPC) injection (*n* = 8 mice). (c) Neutral red (NR) labeling was detected in posterior IC in mice with motor deficit mice at 7 dpl (arrow). Motor deficit was defined as mice exhibiting decreased longest hanging time by more than 60 s. (d) Enlargement of the posterior IC, left panel showing NR‐labeled lesion (outlined), right panel showing the non‐lesioned IC without NR labeling. (e) Partial NR labeling in LPC‐injected IC in a mouse with mild motor deficit. Mild motor deficit was defined as mice exhibiting decreased longest hanging time from 30 s to 60 s. (f) No NR labeling in the IC of a mouse with no motor deficit. No motor deficit was defined as mice exhibiting increased or decreased longest hanging time for less than 30 s. Mean ± *SD*. Student's *t*‐test. ***p < .001

To evaluate IC damage, LPC was stereotactically injected into the right posterior IC of adult mice. Motor behavior was examined by a wire‐hanging task at 7 days post injury (Figure 2a). This task measures the total time a mouse hangs on a stiff wire before falling (Aartsma‐Rus & van Putten, 2014; Dorchies et al., 2013). A mouse without motor deficit is capable of gripping on to and hanging from the wire with all four limbs and maintaining its balance for several minutes. We found that after LPC injection, mice displayed significantly reduced hanging time (Figure 2b; Pre, 211.1 ± 89.09 s; 7 days, 85 ± 33.87 s), suggesting that LPC‐induced IC damage induced motor impairment. To determine if the level of motor deficit corresponded with IC damage, NR labeling, a method to visualize inflammatory white matter lesions (Baydyuk et al., 2019), was performed on mice with motor deficits after LPC injection. We found that mice with significant motor deficit exhibited strong NR labeling on the right medial and posterior IC (Figure 2c,d). In contrast, no NR labeling was observed outside the right IC, or in the contralateral, non‐lesioned IC (Figure 2c,d). Moreover, mice with mild motor deficit exhibited a smaller lesion area and weak NR labeling in the right posterior IC (Figure 2e). We also performed NR labeling on mice without any motor deficit and found that the IC remained intact without any NR labeling, corresponding with the absence of a successful lesion at the IC (Figure 2f). These results suggest that NR is a useful postmortem indicator of successful IC lesion and an important proxy for assessing the extent of IC damage and associated behavioral impairment. We found the percentage of mice that displayed successful IC lesions was approximately 80%. Moreover, we did not encounter situations in which IC demyelination via LPC was not captured by NR labeling, since NR labels activated microglia and reactive astrocytes but not oligodendrocytes under an injury/inflammatory environment (Baydyuk et al., 2019). Taken together, these results suggest that LPC‐induced IC demyelination results in the development of motor deficit in mice.

**Figure 3:**
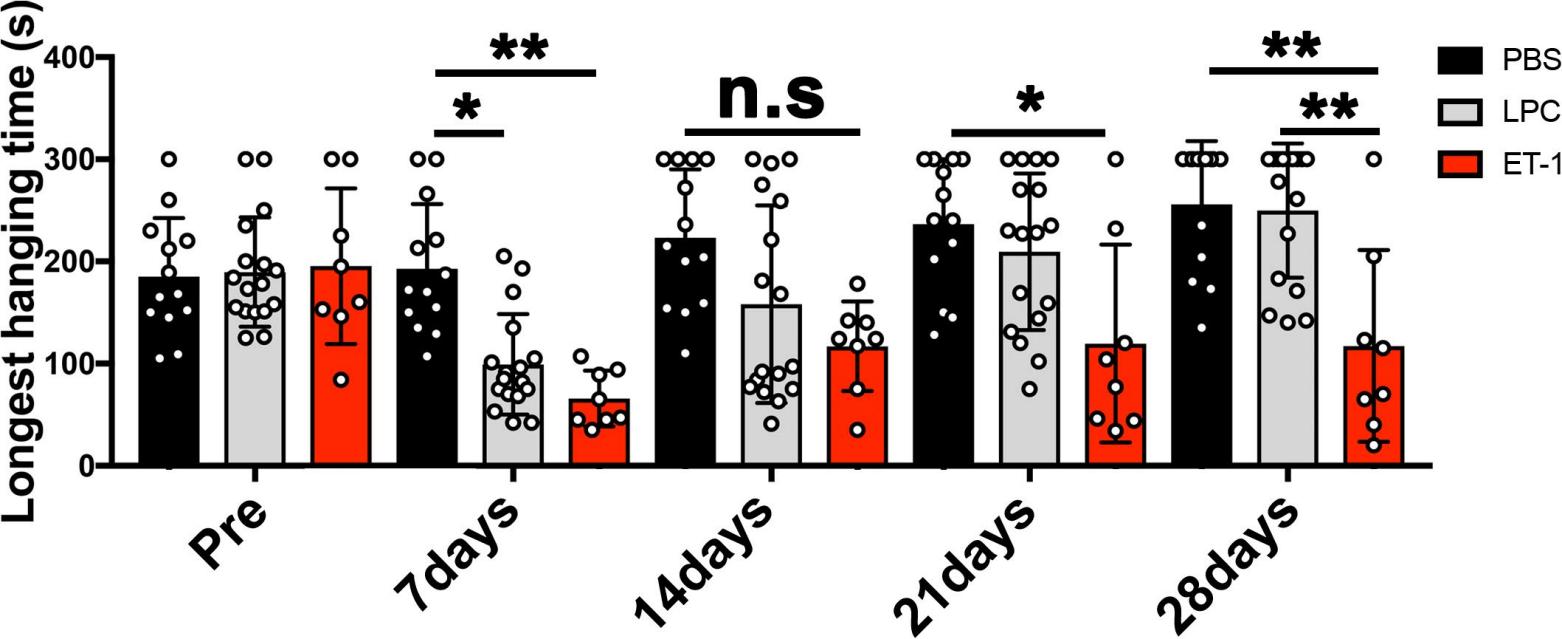
Analysis of motor function between lysophosphatidylcholine (LPC)‐ and endothelin‐1 (ET1)‐induced internal capsule (IC) injuries. Wire‐hanging test showing motor function in the PBS‐injected mice remained intact in all timepoints analyzed. In contrast, LPC‐injected mice displayed motor deficit at 7 and 14 dpl, and gradually recovered by 28 dpl. ET1‐injected mice displayed motor deficit in all post‐lesion timepoints but did not recover (PBS, *n* = 13 mice; LPC, *n* = 17 mice; ET1, *n* = 8 mice). Mean ± *SD*. Two‐way ANOVA, Tukey–Kramer test. *p < .05, **p < .01, n.s., not significant

IC stroke injury is commonly associated with the development of lasting motor deficit, accompanied by a loss of oligodendrocytes, axonal degeneration, inflammation, and fibrosis at the infarct area (Blasi et al., 2015). Given the ability for myelin to regenerate spontaneously after demyelination, we hypothesized that IC demyelination could lead to functional deficit, and subsequent motor recovery through remyelination. To compare motor behavior in IC‐demyelinated lesions to stroke lesions in mice, we performed LPC‐induced demyelination verses endothelin (ET1)‐induced infarct at the right IC, respectively. ET1 is a potent vasoconstrictor, which is commonly used to induce white matter infarction in rodents (Blasi et al., 2015; Roome et al., 2014). As control, PBS was injected into the right IC. Motor behavior was examined by performing a wire‐hanging test on these mice every 7 days for 28 days after injury. We found that mice that received either LPC or ET1 IC injections displayed significantly reduced hanging time at 7 dpl compared to before injury or to PBS injections (Figure 3 and Table S2). Moreover, we found that motor deficit in ET1‐injected mice was detected at all post lesion time points analyzed, suggesting that ET1‐induced IC damage results in lasting motor dysfunction. In contrast, we found that LPC‐injected mice exhibited improved motor function at 14 dpl and recovered to similar functional levels as PBS‐injected mice by 28 dpl (Figure 3). These results suggest that IC demyelination induces similar decline in motor function as IC infarct initially. However, unlike mice with ET1‐induced damage, which do not recover from motor impairment, mice with LPC‐induced damage eventually regain motor function by 28 dpl.

### 3.2 Recovery of motor behavior following LPC‐induced IC damage is associated with increased oligodendrocytes and myelin and decreased axonal dystrophy

**Figure 4:**
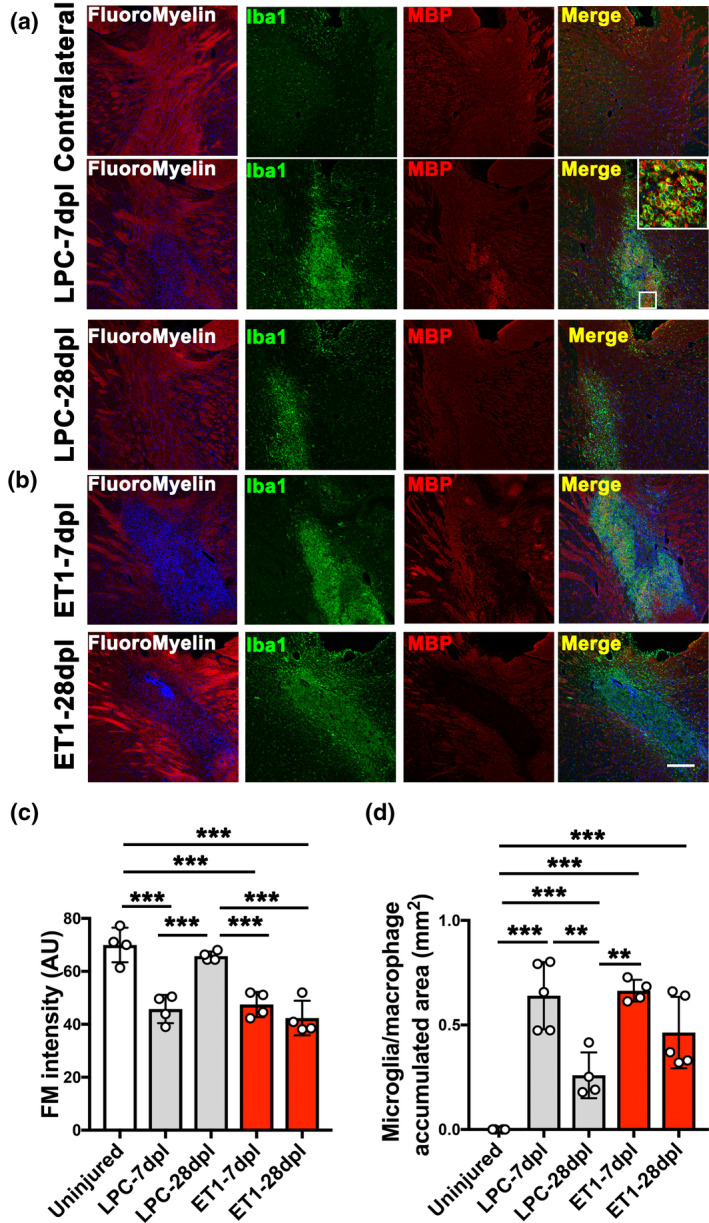
Analysis of myelin staining and inflammation in internal capsule (IC) lesions. (a, b) FluoroMyelin staining (red) and double immunofluorescence images of lysophosphatidylcholine (LPC)‐lesioned IC at 7 and 28 dpl, and endothelin‐1 (ET1)‐lesioned IC at 7 and 28 dpl labeled with anti‐ionized calcium‐binding adapter molecule 1 (Iba1) (green) and anti‐myelin basic protein (MBP) (red) antibodies. Inset shows an enlarged image of myelin fragments in Iba1^+^ microglia/macrophages. Nuclei were counterstained with Hoechst (blue). Scale bar, 200 µm. (c) Quantification of fluorescence intensity based on FluoroMyelin (FM) staining in uninjured contralateral IC, LPC‐lesioned IC, and ET1‐lesioned IC at 7 and 28 dpl (d) Quantification of Iba1^+^ microglia/macrophage accumulation in uninjured contralateral IC, LPC‐lesioned IC, and ET1‐lesioned IC at 7 and 28 dpl (LPC‐7 dpl, ET1‐28 dpl *n* = 5 mice; LPC‐28 dpl, ET1‐7 dpl *n* = 4 mice). Mean ± *SD*. One‐way ANOVA, Tukey–Kramer test. **p < .01, ***p < .001

To examine the state of myelination and inflammation after IC injury, we performed Fluoromyelin staining as well as MBP and Iba1 co‐labeling on CNS tissue sections from lesioned mice after motor behavioral tasks were performed at 7 and 28 dpl. In LPC‐lesioned mice, we detected a significant loss of Fluoromyelin staining at 7 dpl, corresponding to demyelination, when compared to the contralateral uninjured IC (Figure 4a,c). Moreover, intense Iba1 labeling, corresponding with increased activated microglia/macrophage density, was observed at lesions devoid of Fluoromyelin staining (Figure 4a,d). The enhanced Iba1 staining was not detected in the contralateral IC or in PBS‐injected IC (Figure 4a,d and Figure S1a). Moreover, within LPC‐injected lesions, we observed clusters of MBP labeling at the vicinity of or inside Iba1^+^ cells, suggesting that myelin fragments were phagocytosed by activated microglia/macrophages (Figure 4a, Inset). At 28 days after LPC injection, we found that Fluoromyelin and MBP staining at the IC appeared to have returned to similar level as non‐lesioned or PBS‐injected IC, suggesting that remyelination had occurred by this timepoint (Figure 4a,c and Figure S1b). Moreover, we detected a significant decrease in Iba1 accumulation in lesions at 28 dpl compared to 7 dpl, suggesting that inflammation was reduced during remyelination (Figure 4a,d). We also examined the extent of IC damage at 7 and 28 days after ET1 injection (Figure 4b). We found that mice that received ET1 injections displayed reduced Fluoromyelin and MBP staining, and enhanced Iba1 staining in the IC at 7 dpl (Figure 4b), similarly to those that received LPC injections (Figure 4c,d). However, in contrast to LPC‐induced lesions, Fluoromyelin/MBP staining remained reduced at 28 dpl in ET1‐induced lesions and did not return to control levels (Figure 4b,c). Moreover, accumulation of Iba1^+^ microglia/macrophages remained elevated in ET1 lesions at 28 dpl (Figure 4b,d). These results suggest that ET1 injection led to prolonged inflammation and myelin loss at the IC, whereas LPC injection resulted in inflammation resolution and remyelination by 28 dpl, which might have contributed to the observed functional recovery.

**Figure 5:**
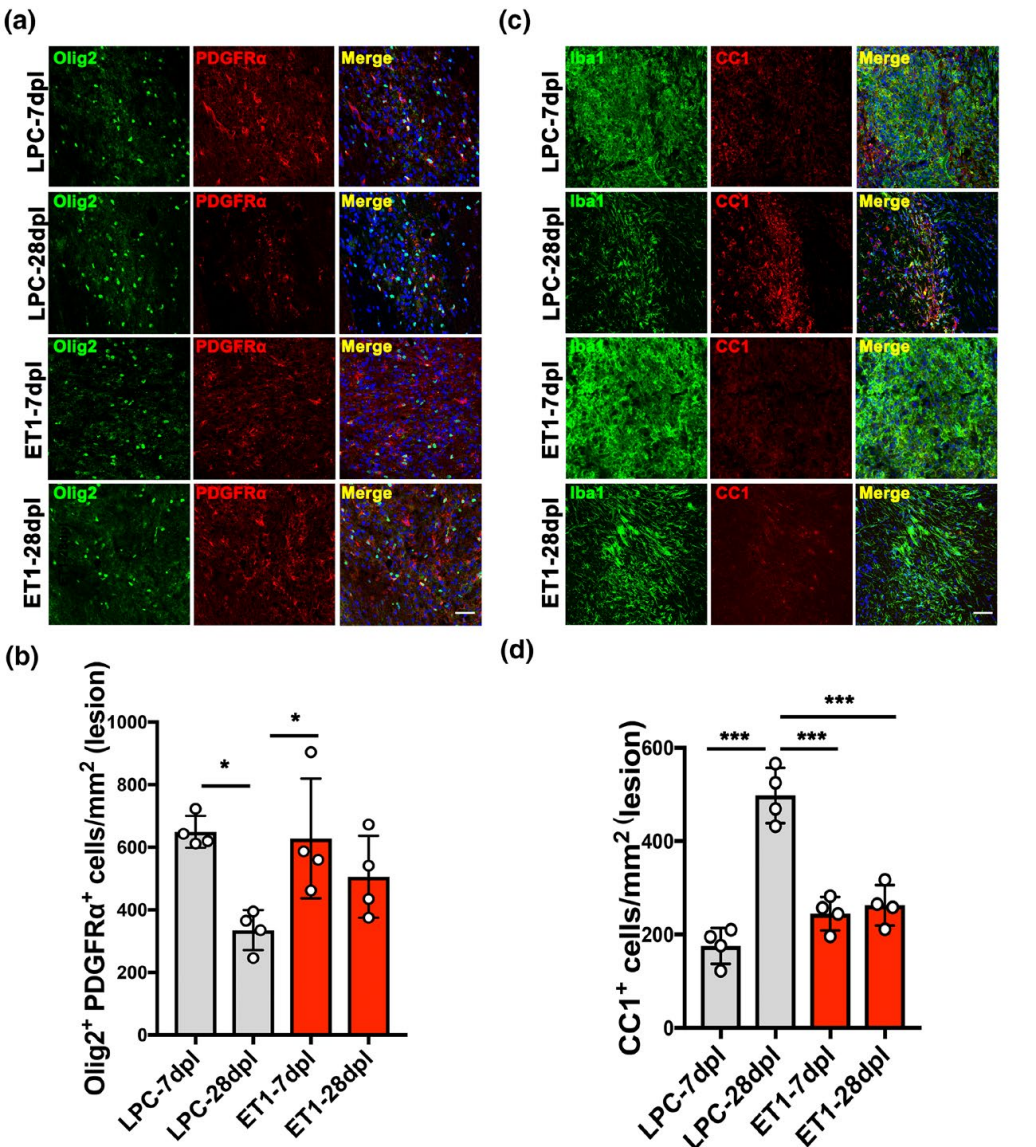
Analysis of oligodendrocyte lineage cells in internal capsule (IC) lesions. (a) Double immunofluorescence images of lysophosphatidylcholine (LPC)‐lesioned IC‐ and endothelin‐1 (ET1)‐lesioned IC at 7 and 28 dpl labeled with anti‐Olig2 (green) and anti‐platelet‐derived growth factor receptor α chain (PDGFRα) (red) antibodies. Nuclei were counterstained with Hoechst (blue). Scale bar, 50 µm. (b) Quantification of the density of Olig2 and PDGFRα double‐positive cells in LPC‐ versus. ET1‐induced IC lesions at 7 and 28 dpl (*n* = 4 mice/group). (c) Double immunofluorescence images of LPC‐lesioned IC and ET1‐lesioned IC at 7 and 28 dpl labeled with anti‐ionized calcium‐binding adapter molecule 1 (green) and anti‐CC1 (red) antibodies. Nuclei were counterstained with Hoechst (blue). Scale bar, 50 µm. (d) Quantification of CC1‐positive cells in LPC‐ versus. ET1‐induced IC lesions at 7 and 28 dpl (*n* = 4 mice/group). Mean ± *SD*. One‐way ANOVA, Tukey–Kramer test. *p < .05, ***p < .001

**Figure 6:**
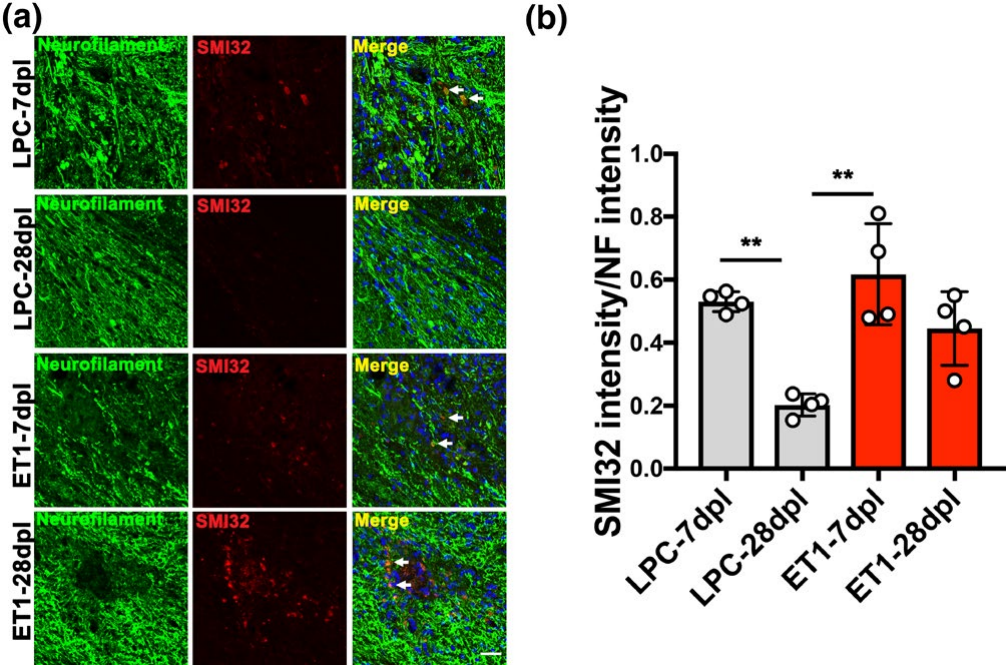
Analysis of axonal dystrophy in internal capsule (IC) lesions. (a) Double immunofluorescence images of lysophosphatidylcholine (LPC)‐lesioned IC‐ and endothelin‐1 (ET1)‐lesioned IC at 7 and 28 dpl labeled with anti‐neurofilament (NF) (green) and anti‐SMI32 (red) antibodies. Nuclei were counterstained with Hoechst (blue). SMI32‐positve axons were observed in LPC lesions at 7 dpl, and ET1 lesions at 7 and 28 dpl (arrow). Scale bar, 20 µm. (b) Quantification of fluorescence intensity of SMI32 to NF (*n* = 4 mice/group). Mean ± *SD*. One‐way ANOVA, Tukey–Kramer test. **p < .01

During remyelination, oligodendrocyte precursor cells (OPCs) are known to migrate to demyelinated lesions and differentiate into mature myelinating oligodendrocytes (OLs) (Franklin & ffrench‐Constant, 2008). To examine the number of OPCs in LPC‐ versus ET1‐induced IC lesions, immunostaining analysis for PDGFRα and Olig2 co‐labeling was performed on CNS sections at 7 and 28 dpl. We found the density of PDGFRα^+^Olig2^+^ OPCs in LPC‐induced lesions decreased significantly from 7 dpl (651.4 ± 44.14 cells/mm^2^) to 28 dpl (335.3 ± 78.81 cells/mm^2^), which might correspond to oligodendrocyte differentiation prior to remyelination (Figure 5a,b). OPCs were also observed in ET1‐induced lesions at 7 dpl (628.3 ± 191.5 cells/mm^2^), but their relative density in lesions was unchanged at 28 dpl (506 ± 130.8 cells/mm^2^) (Figure 5a,b). To examine the density of mature OLs in IC lesions, immunostaining analysis for CC1 was performed. Additionally, co‐labeling with Iba1 was performed to localize the lesion. We found that the density of CC1^+^ OLs in LPC‐induced lesions increased significantly from 7 dpl (175.8 ± 30.58 cells/mm^2^) to 28 dpl (498.0 ± 59.3 cells/mm^2^), suggesting that OPCs recruited to the lesions have differentiated into myelinating OLs at 28 dpl (Figure 5c,d). Moreover, Iba1 staining and inducible nitric oxide synthase‐positive proinflammatory microglia/macrophage staining were reduced significantly from 7 to 28 dpl, suggesting an association between inflammation resolution and oligodendrocyte regeneration in LPC‐induced lesions (Figure S2). In contrast, the density of CC1^+^ OLs in ET1‐induced lesions did not change significantly from 7 dpl (244.8 ± 36.12 cells/mm^2^) to 28 dpl (262.8 ± 43.39 cells/mm^2^) (Figure 5c,d). Moreover, we found that mice with ET1 lesions displayed significantly fewer CC1^+^ OLs (262.8 ± 43.39 cells/mm^2^) compared to LPC lesions at 28 dpl, suggesting that OL differentiation was impaired (Figure 5d). Next, to investigate the extent of axonal dystrophy in LPC and ET1‐induced lesions, immunofluorescence analysis for SMI32 and NF was performed. We detected SMI32^+^NF^+^ labeling in LPC‐induced lesions at 7 dpl (0.5308 ± 0.0317), but this was significantly reduced at 28 dpl (0.2018 ± 0.04329) (Figure 6a,b). In contrast, SMI32^+^NF^+^ labeling remained detectable in ET1‐induced lesions from 7 dpl (0.6175 ± 0.1607) to 28 dpl (0.4433 ± 0.1436) (Figure 6a,b), suggesting that ET1 injection resulted in lasting oligodendrocyte/myelin loss and axonal dystrophy. Together, these results suggest that inflammation resolution and remyelination promoted motor recovery after LPC‐induced IC demyelination.

### 3.3 LPC‐induced IC damage results in asymmetric forelimb and hindlimb motor impairment

**Figure 7:**
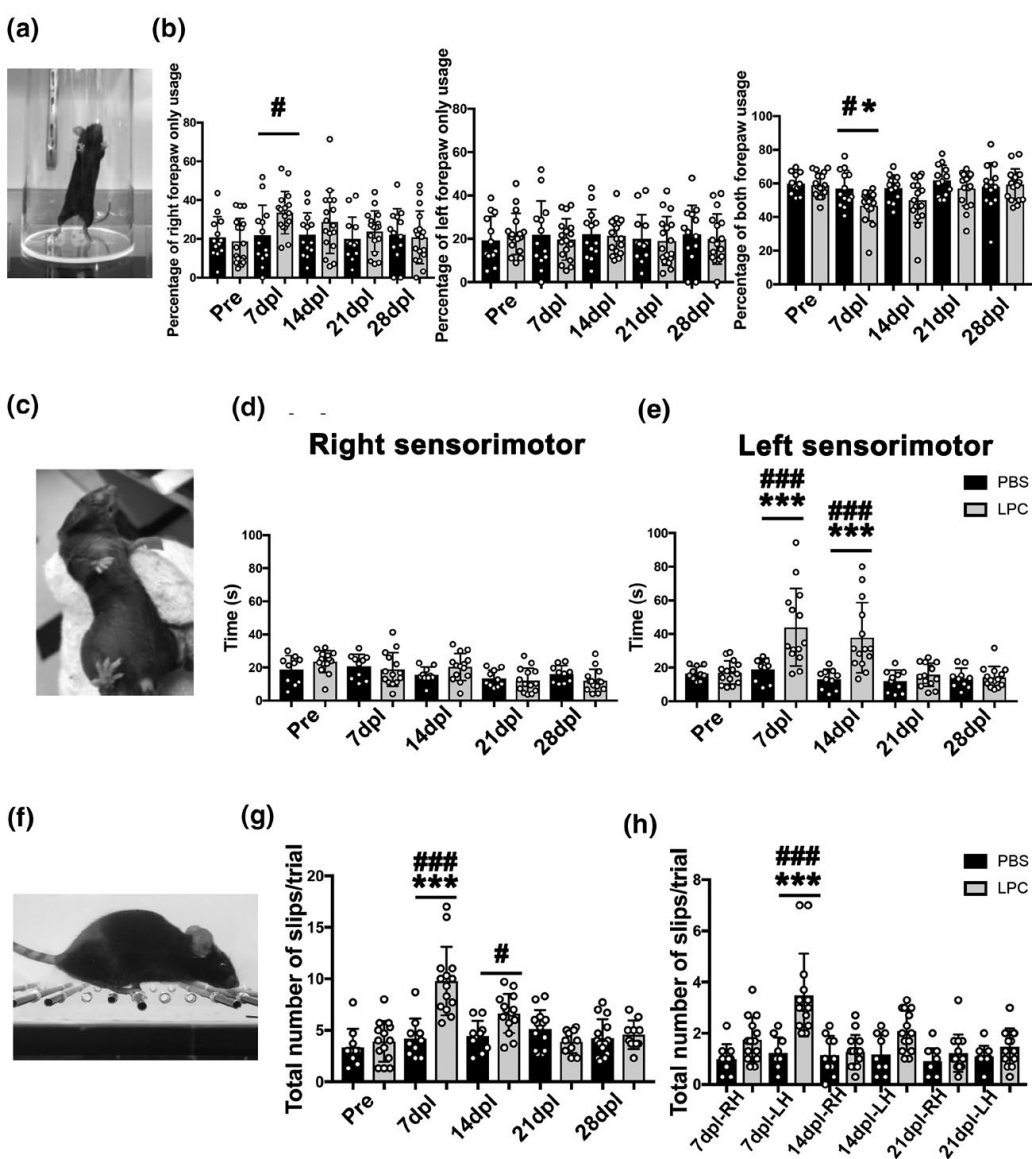
Cylinder test, adhesive tape removal test, and ladder test after lysophosphatidylcholine (LPC)‐induced internal capsule (IC) lesion. (a) Image of a mouse performing the cylinder test. (b) Quantification of the ratio of right only, left only, or both forepaw usage (PBS, *n* = 13 mice; LPC, *n* = 17 mice). (c) Adhesive tape removal test by application of a sticky tape to the mouse forepaw. (d) Right (ipsilateral) sensorimotor scores based on time of tape contact and removal for each forepaw following right IC lesion show no significant difference between PBS‐ and LPC‐injected mice (PBS, *n* = 10 mice; LPC, *n* = 14 mice). (e) Left (contralateral) sensorimotor scores show significant increase in time of tape removal in LPC‐injected mice compared to PBS injection. (f) Image of a mouse performing the ladder test. (g) The total number of foot slips in LPC‐injected mice increased significantly at 7 dpl compared to PBS‐injected mice, but the numbers gradually returned to control level by 28 dpl. (h) Analysis of right versus. left hindlimb slips shows increased slips in the left hindlimb at 7 and 14 dpl (PBS, *n* = 10 mice; LPC, *n* = 14 mice). Mean ± *SD*. Two‐way ANOVA, Tukey–Kramer test. *p < .05, ***p < .001 LPC versus. PBS; ^#^ *p* < .05, ^###^ *p* < .001 LPC versus. Pre (baseline)

Unilateral IC damage has been shown to induce asymmetric motor disability in stroke and MS patients (Bitsch, Schuchardt, Bunkowski, Kuhlmann, & Brück, 2000; Lee et al., 2000). Since the corticospinal tract regulates the movement of contralateral limbs, we next asked if LPC‐induced demyelination results in asymmetric motor deficit and recovery in mice. We examined forepaw motor function by performing a cylinder test on mice that have received either PBS or LPC injection in the right IC. The cylinder test is an exploratory behavioral test that is frequently used to assess forepaw preference use in rodent experimental lesion or neurodegeneration models (Blasi et al., 2015; Starkey et al., 2005). For this task, mice were placed in a clear cylinder (Figure 7a and Figure S3a–c), and the total number of cylinder touches, corresponding to weight support during exploratory behavior, using right forepaw only (RFO), left forepaw only (LFO), or both forepaws, was filmed and counted for 7 min. Prior to IC lesion, we found that mice contacted the cylinder with both forepaws approximately 60% of the time, indicating a preference for using both forepaws during exploration (Figure 7b). These mice also used RFO and LFO contacts equally, at approximately 20% of the time for each forepaw, indicating no preference for a particular paw. In PBS‐injected mice, we found the percentages of forepaw use or preference remained unchanged throughout all the timepoints analyzed (Figure 7b and Table S3). In contrast in the LPC‐injected mice, the percentage of both forepaw use was reduced significantly at 7 dpl. Moreover, RFO usage increased significantly. The use of both forepaws and RFO use returned to control levels by 28 dpl along with other measures of functional motor recovery. The percentage of LFO usage remained constant at all timepoints analyzed. These results suggest that right IC damage results in asymmetric motor deficit and a dependence on the use of RFO contact over the left forepaw.

To examine possible sensorimotor dysfunction in IC‐demyelinated mice, an adhesive tape removal test was performed on PBS and LPC IC‐lesioned mice. The adhesive tape removal test allows the assessment of sensorimotor deficit after the placement of a small piece of adhesive tape onto the surface of a forepaw of a mouse that has received an experimental CNS lesion (Figure 7c) (Bouet et al., 2009; Starkey et al., 2005; Tennant & Jones, 2009). Sensorimotor deficit scores are determined by the total time it takes for the mouse to remove the tape from its forepaw, either through shaking the tape off or removing it with its mouth, after being placed in the cage. Following training, we recorded the sensorimotor scores of each forepaw from mice before injury during three trials, and after PBS or LPC injection at 7, 14, 21, and 28 dpl. In the PBS‐injected mice, we found that both the left and right sensorimotor scores did not change significantly compared to pre‐injury scores and remained relatively constant across all timepoints analyzed (Figure 7d,e and Table S4). In contrast, the LPC‐injected mice exhibited asymmetric behavioral deficit at 7 and 14 dpl, in which the sensorimotor scores of the left forepaw showed motor deficits, indicated by significantly increased times, whereas right forepaw scores remained similar to control animals. By 21 dpl, the left forepaw scores returned to control level, indicating a recovery of sensorimotor function (Figure 7d,e and Table S4). These results suggest that IC demyelination results in transient asymmetric sensorimotor impairment and eventual recovery.

To evaluate motor disability in both fore‐ and hindlimbs after LPC injection, we next performed a horizontal ladder test (Figure 7f and Figure S4d–f) (Farr et al., 2006; Metz & Whishaw, 2002). For this task, mice walk on a horizontally placed 120cm ladder with irregularly spaced rungs from a start cage toward a home cage, and the total number of foot slips on the ladder is filmed and counted during three trials. We found the PBS‐injected mice performed the ladder walking test similarly to mice before IC injury, suggesting normal motor function. In contrast, LPC‐injected mice displayed a significant increase in the number of foot slips at 7 dpl, but these mice gradually recovered between 14 and 28 dpl (Figure 7g and Table S5). To examine hindlimb motor disability, we quantified the total number of foot slips individually in the right hindlimb and left hindlimb (LH). Compared to PBS‐injected mice, LPC‐injected mice exhibited motor disability at 7 dpl in both right hindlimb and LH, with the LH displaying significantly greater motor deficit (Figure 7h and Table S5). Moreover, LH motor deficit remained detectable in the LPC‐injected mice at 14 dpl. By 21 dpl, motor function in LPC‐injected mice returned to similar level as PBS‐injected mice, suggesting a recovery from motor deficit by this time. Together, these results suggest that focal IC demyelination leads to asymmetric motor deficit in both fore‐ and hindlimbs.

## 4 DISCUSSION

Toxin‐induced demyelination in the corpus callosum and spinal cord have been frequently used in remyelination studies. However, mice from these models do not display obvious or measurable motor deficit. In this study, we found that focal injection of LPC into the IC‐induced asymmetric acute motor deficit and subsequent motor recovery. Our results suggest that inflammation resolution and remyelination may be required for restoring axonal integrity and motor recovery. Interestingly, it has previously been shown in cats that the ingestion of irradiated diet induces global CNS demyelination and profound functional deficit, and the return to unirradiated diet results in extensive remyelination and functional recovery (Duncan, Brower, Kondo, Curlee, & Schultz, 2009). Our study in mice show that under an injury‐induced demyelination environment, inflammation resolution and remyelination plays a critical role in functional motor recovery.

The posterior IC houses the corticospinal tract that regulates contralateral limb movement. To determine if IC damage produces motor deficit, we injected LPC at several stereotactic points at the posterior IC. We found that substantial focal demyelination at the posterior IC was needed to induce a measurable locomotor deficit. Indeed, injection of LPC into the posterior IC resulted in asymmetric motor disability at 7 dpl by wire‐hanging test, cylinder test, adhesive tape removal test, and horizontal ladder test. The motor deficit remained at 14 dpl, but these mice eventually recovered by 28 dpl. The severity of motor deficit depended on the demyelinated‐lesion size and position at the IC, which could be identified through neutral red labeling. For example, mice with detectable motor deficit displayed a large lesion at the posterior IC, whereas those with no detectable deficit displayed a small IC lesion, or a lesion outside of the posterior IC. Therefore, checking the size and position of IC lesion after performing the behavioral tasks is important to ensure reproducibility of the behavioral studies.

Previous reports have determined that acute axonal injury or loss correlates with motor impairment and paralysis in MS patients with internal capsule damage (Bitsch et al., 2000; Lee et al., 2000). We found that acute IC demyelination in mice by LPC injections induced axonal dystrophy at 7 dpl, but this was reduced by 28 dpl. In contrast, ET1‐induced lesion resulted in lasting motor deficit, corresponding to prolonged inflammation, remyelination failure, and sustained axonal injury. Together, these results suggest that decreased inflammation and remyelination in lesions may have contributed to the recovery of axonal integrity and motor behavior in LPC‐injected mice.

Although ET1 is a well‐known vasoconstrictor, and used as an agent to induce stroke in rodents, it is also highly expressed by reactive astrocytes during demyelination and known to promote astrocyte proliferation (Gadea, Schinelli, & Gallo, 2008) and inhibit remyelination (Hammond et al., 2014). Previous study showed that ET1 infusion prevented OPC differentiation into mature oligodendrocytes by promoting Notch activation in OPCs through induction of Jagged expression in reactive astrocytes (Hammond et al., 2014). However, given the rapid clearance and short half‐life of ET1 (plasma half‐life of 40 s) (Sirviö, Metsärinne, Saijonmaa, & Fyhrquist, 1990), it is likely that ET1 injection induced inflammation and myelin loss through vasoconstriction and infarct formation at the IC. However, it remains possible that the level of recovery in ET1‐treated mice might depend on the concentration of ET1 delivered, which could affect the severity of vasoconstriction and remyelination efficiency. Future studies would be necessary to determine if different ET1 concentrations affect the extent of oligodendrocyte lineage cell progression and remyelination. Moreover, it would be important to determine the potentially different pathologies between the ET1 and LPC models by examining the early stage of injury. We have found that ET1‐induced lesions at 3 dpl displayed higher fibrosis level (based on collagen type1 expression) compared to LPC‐induced lesions (Figure S4), suggesting that ET1‐induced vasoconstriction might have interfered with the remyelination process through increased fibrosis in lesions. Moreover, it remains possible that ET1‐induced pathology differs from LPC‐induced pathology by affecting neuronal integrity rather than directly on myelin/oligodendrocytes. In conclusion, LPC‐induced IC demyelination induces transient motor deficit and subsequent functional recovery through remyelination. Our results suggest that IC demyelination may be a promising model for assessing the influence of remyelination on motor behavior, which may be used to complement future drug screens for the identification of remyelination compounds in MS.

## CONFLICT OF INTEREST

The authors declare no competing financial interests.

## AUTHOR CONTRIBUTIONS

J.K.H. conceived and oversaw the study. J.K.H. and R.Y. designed the study. R.Y. established the mouse IC demyelination strategy and set up behavioral tasks and measurements. R.Y. performed all experiments and data analyses. J.K.H., N.O., and R.Y. wrote the manuscript. All the authors approved the final version of the manuscript.

## Supporting information

Supplementary Material
