# Supplementary material for: Acute motor deficit and subsequent remyelination‐associated recovery following internal capsule demyelination in mice
Source: J Neurochem. 2020 Aug 14;156(6):917–28. doi: 10.1111/jnc.15142 (PMC8048697; doi:10.1111/jnc.15142)
Supplement: Supplementary file 1 — Supplementary Material [file JNC-156-917-s001.pdf]

Supporting information

Acute motor deficit and subsequent remyelination-associated recovery following internal capsule demyelination in mice

Short title: *Analysis of motor function in mice after internal capsule demyelination*

Reiji Yamazaki[1,2], Nobuhiko Ohno[2] and Jeffrey K. Huang[1,3]

[1]Department of Biology and Center for Cell Reprogramming, Georgetown University, Washington, DC 20057, USA

[2]Department of Anatomy, Division of Histology and Cell Biology, School of Medicine, Jichi Medical University, Shimotsuke, Japan ：

[3]Correspondence should be addressed to J.K.H. (jh1659@georgetown.edu)

This file includes:
Tables S1-S5
Figures S1-S4

Supplementary Tables

Table S1. Mice used in this study

	Injection Type	Task	Timepoints Analyzed	Total Mice Used	Sac'd at 3dpl for Immuno	Sac'd at 7dpl for Immuno	Sac'd at 28dpl for immuno	Wire Hanging/ Cylinder Tests	Tape Removal/ Ladder Tests	Excluded	Figure
**Initial Testing**	LPC	Wire Hang/NR	Pre and 7dpl	8		8					**Fig 2**
**Immuno/ Behavior**	LPC	Behavior/Immuno	Pre, 7, 14, 21, 28dpl	39	3	5	5 (after behavioral test)	17	14		**Fig 3, 7 (behavior); Fig. 4, 5, 6, S4 (immuno)**
	PBS	Behavior/Immuno	Pre, 7, 14, 21, 28dpl	23	0	0	5 (after behavioral test)	13	10		**Fig 3, 7 (behavior); Fig. 4, 5, 6, S4 (immuno)**
	ET1	Behavior/Immuno	Pre, 7, 14, 21, 28dpl	18	3	5	5 (after behavioral test)	8		2	**Fig 3, 7 (behavior); Fig. 4, 5, 6, S4 (immuno)**
**Total Mice**				88							

Table S2. Wire hanging test

Hanging time (seconds)	PBS	LPC	ET1
Pre	185.0 ± 53.42	189.6 ± 57.46	195.4 ± 82.32
7dpl	192.7 ± 49.10	99.29 ± 63.62	66.0 ± 29.43
14dpl	223.1 ± 96.69	158.30 ± 67.20	116.9 ± 47.37
21dpl	236.5 ± 76.69	209.40 ± 63.69	119.6 ± 104.60
28dpl	255.9 ± 65.69	249.90 ± 61.87	127.6 ± 101.30

Graph indicated the mean ± standard deviation (SD) obtained from each eight mice (PBS; n=13, LPC; n=17, ET1; n=8). Asterisks indicate *P<0.05, **P<0.01. ***P<0.001 versus PBS, $^{\#}$P<0.05 versus Pre (baseline) by Tukey-Kramer test after two-way ANOVA.

Table S3. Cylinder test

LFO (sec)	Pre	7dpl	14dpl	21dpl	28dpl
PBS	19.33 ± 11	21.15 ± 11.24	20.57 ± 11.82	18.31 ± 11.43	19.82 ± 20.17
LPC	22.25 ± 9.544	19.71 ± 9.514	21.32 ± 8.358	19.04 ± 11.2	19.88 ± 11.61

RFO (sec)	Pre	7dpl	14dpl	21dpl	28dpl
PBS	20.78 ± 10.64	21.84 ± 15.57	22.10 ± 11.41	20.02 ± 11.18	22.05 ± 13.48
LPC	18.79 ± 11.7	33.55 ± 10.88	28.74 ± 16.32	23.89 ± 10.55	20.77 ± 13.58

Both (sec)	Pre	7dpl	14dpl	21dpl	28dpl
PBS	59.88 ± 7.872	57.01 ± 10.74	57.33 ± 7.667	61.67 ± 9.107	58.13 ± 13.98
LPC	58.95 ± 7.05	46.75 ± 9.045	49.95 ± 13.26	57.06 ± 2.506	59.35 ± 9.388

Quantification of the ratio of right or left or both forepaw usage. Graph indicated the mean ± standard deviation (SD) obtained from each group (PBS; n=13, LPC; n=17). Two-way ANOVA, Tukey-Kramer test. $^{*}P<0.05$ versus PBS. $^{\#}P<0.05$ versus Pre (baseline).

Table S4. Adhesive tape removal test

Right-motor (sec)	Pre	7dpl	14dpl	21dpl	28dpl
PBS	18.75 ± 8.439	20.70 ± 7.364	15.55 ± 4.728	13.42 ± 5.076	16.02 ± 5.094
LPC	23.61 ± 6.599	18.84 ± 10.22	20.36 ± 8.154	12.24 ± 7.354	12.09 ± 6.825

Left-motor (sec)	Pre	7dpl	14dpl	21dpl	28dpl
PBS	16.65 ± 4.079	18.97 ± 6.719	13.15 ± 5.892	12.07 ± 6.467	13.68 ± 5.952
LPC	17.14 ± 6.895	43.96 ± 23.07	37.84 ± 20.76	15.80 ± 6.918	14.23 ± 6.415

Graph indicated the mean ± standard deviation (SD) obtained from each group (PBS; n=10, LPC; n=14). Two-way ANOVA, Tukey-Kramer test. ***P<0.001 versus PBS. $^{###}$P<0.001 versus Pre (baseline).

Table S5. Horizontal ladder test

Total slips	Pre	7dpl	14dpl	21dpl	28dpl
PBS	3.34 ± 1.807	4.21 ± 1.931	4.44 ± 1.483	3.89 ± 1.122	4.57 ± 1.382
LPC	3.92 ± 1.983	9.78 ± 3.323	6.64 ± 1.896	5.12 ± 1.831	4.24 ± 1.829

Graph indicated the mean ± standard deviation (SD) obtained from each group (PBS; n=10, LPC; n=14).

Hindlimb	PBS	LPC
7dpl-RH	0.99 ± 0.5801	1.75 ± 0.8645
7dpl-LH	1.23 ± 0.6075	3.49 ± 1.618
14dpl-RH	1.14 ± 0.756	1.26 ± 0.6675
14dpl-LH	1.17 ± 0.7558	2.09 ± 0.7995
21dpl-RH	0.90 ± 0.5185	1.22 ± 0.7298
21dpl-LH	1.09 ± 0.4202	1.479 ± 0.7138

Graph indicated the mean ± standard deviation (SD) obtained from each group (PBS; n=10, LPC; n=14). two-way ANOVA, Tukey-Kramer test. $^{***}P<0.001$ versus PBS. $^{###}P<0.001$ versus RH (baseline).

Supplementary Figures

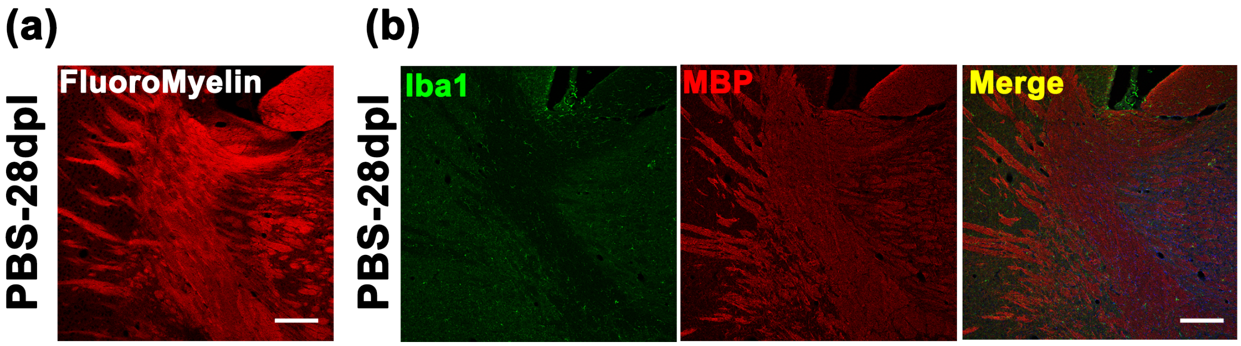

Figure S1. PBS injected IC at 28 dpl. (a) PBS injected IC at 28dpl shows intact FluoroMyelin staining (red). Scale bar: 200 µm. (b) Double immunofluorescence using anti-Iba1 (green) and anti-MBP (red) antibodies shows undisturbed IC. Nuclei are counterstained with Hoechst (blue). Scale bar: 200 µm.

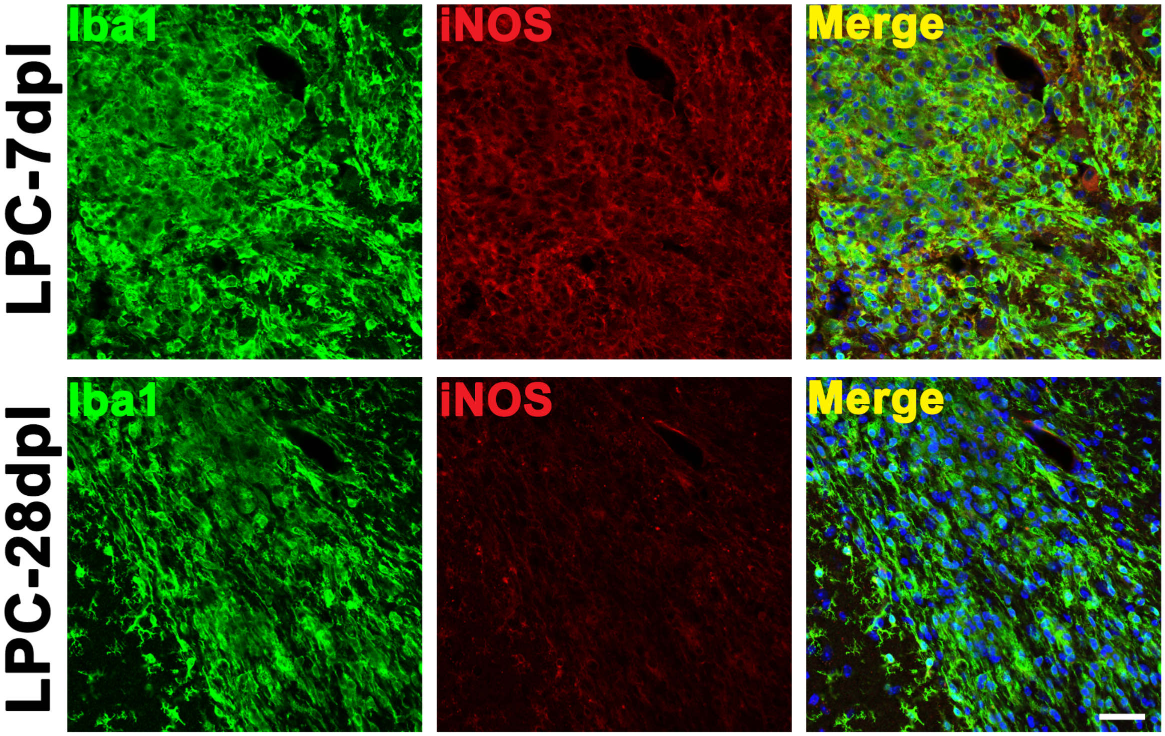

Figure S2. Proinflammatory microglia/macrophage staining in LPC demyelinated lesion. Double immunofluorescence images of brain sections of LPC-7dpl and LPC-28dpl using anti-Iba1 (green) and anti-iNOS (red) antibodies. Nuclei are counterstained with Hoechst (blue). Scale bar: 20 μm.

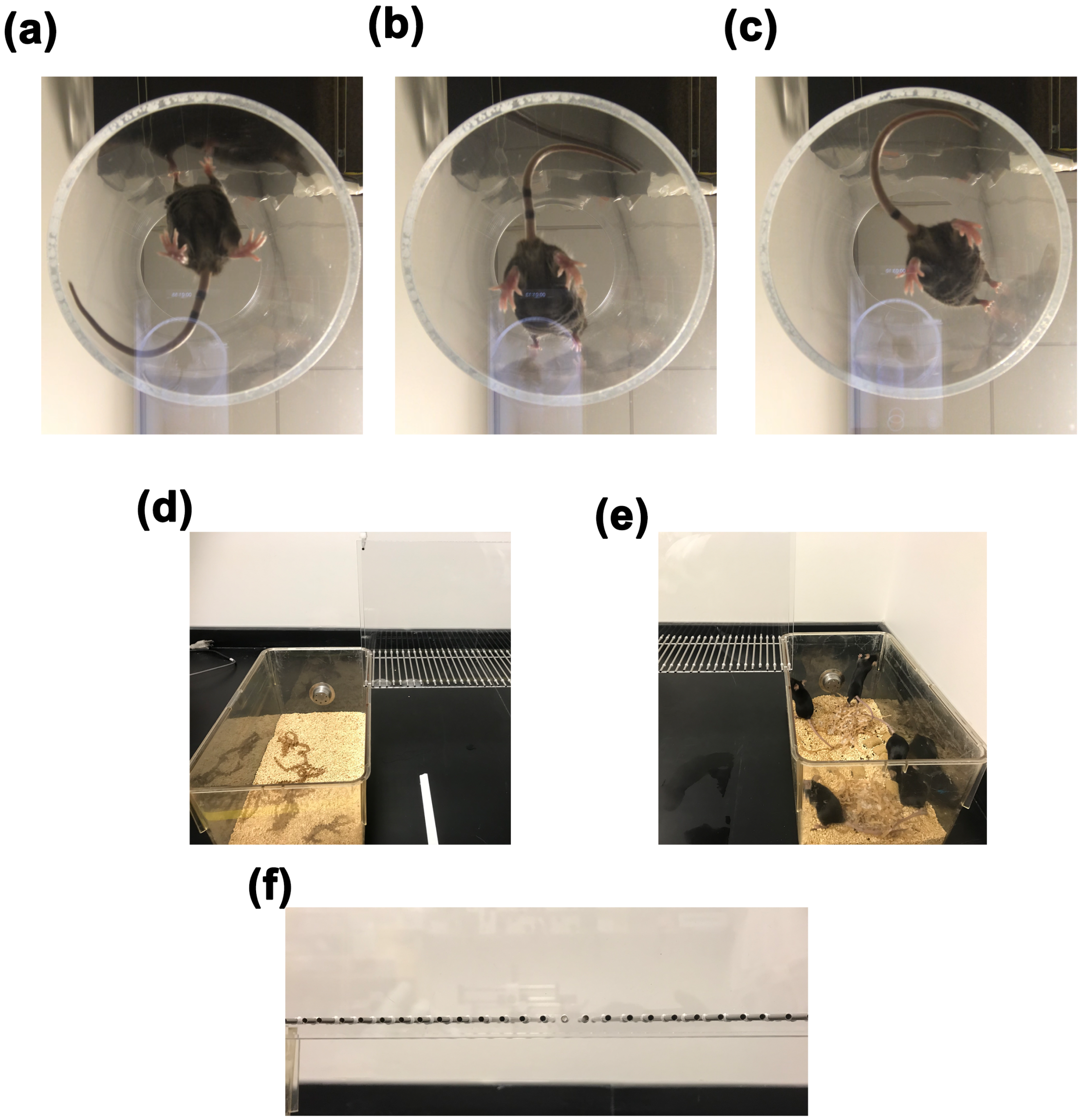

Figure S3. Behavioral tests. (a-c) Cylinder test (a) Representative picture of mouse using its right forepaw for weight support. (b) Representative picture of mouse using its left forepaw for weight support. (c) Representative picture of mouse using both forepaws for weight support. (d-f) Horizontal ladder test (d) Starting cage of ladder (e) Home cage of ladder (f) The picture of the horizontal ladder bar. When mice walk on the ladder, bars were changed to irregularly steps.

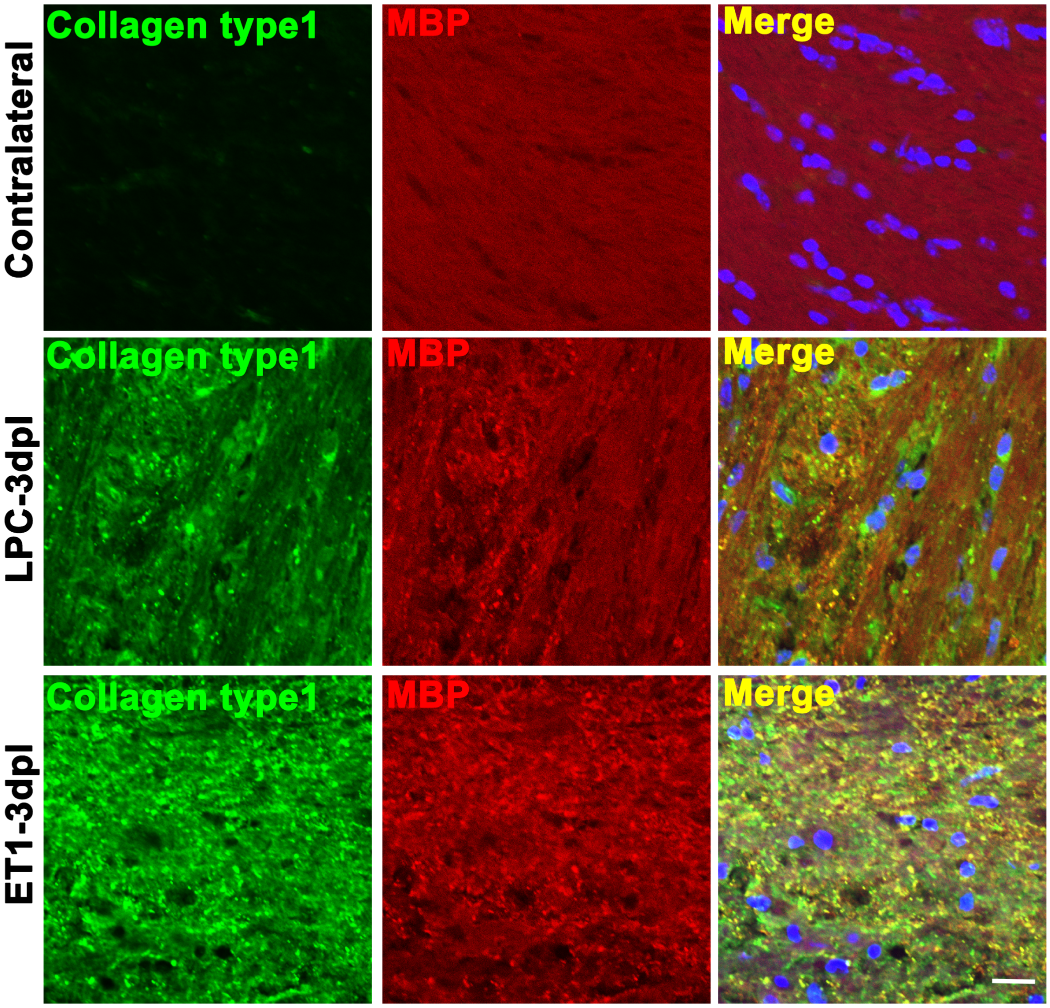

Figure S4. Fibrosis staining in LPC and ET1 lesion. Double immunofluorescence images of brain sections of LPC-3dpl and ET1-3dpl using anti-Collagen type1 (green) and anti-MBP (red) antibodies. Nuclei are counterstained with Hoechst (blue). Scale bar: 20 μm.
